# Supplementary material for: Heidelberg colorectal data set for surgical data science in the sensor operating room
Source: Sci Data. 2021 Apr 12;8:101. doi: 10.1038/s41597-021-00882-2 (PMC8042116; doi:10.1038/s41597-021-00882-2)
Supplement: Supplementary file 1 — Supplementary Material [file 41597_2021_882_MOESM1_ESM.pdf]

Supplementary Information Guide

Supplementary Methods (p. 3-9)

The Supplementary Methods contain the labeling protocol for the Robust Medical Instrument Segmentation (ROBUST-MIS) Challenge 2019.

Supplementary Tables (p. 10)

Supplementary Table 1 (p. 10)

Supplementary Table 1 shows an example of the device data as it is provided in a .csv file for all 30 surgical procedures.

Supplementary Table 2 (p. 11)

Supplementary Table 2 shows an example of the phase annotations as they are provided in a .csv file for all 30 surgical procedures.

Supplementary Information

Heidelberg colorectal data set for surgical data science in the sensor operating room

Maier-Hein et al.

Supplementary Methods

ROBUST-MIS Labeling Instructions

Introduction

Intraoperative tracking of laparoscopic instruments is often a prerequisite for computer and robotic assisted interventions. Although previous challenges have targeted the task of detecting, segmenting and tracking medical instruments based on endoscopic video images, key issues remain to be addressed:

- <u>Robustness</u>: The methods proposed still tend to fail when applied to challenging images (e.g. in the presence of blood, smoke or motion artifacts, different and new instruments).
- <u>Generalization</u>: Algorithms trained for a specific intervention in a specific hospital typically do not generalize.

The goal of this challenge is, therefore, the benchmarking of medical instrument detection and segmentation algorithms, with a specific emphasis on robustness and generalization capabilities of the methods. The challenge is based on the biggest annotated dataset made (to be made) publically available, comprising 10,000 annotated images that have been extracted from a total of 30 surgical procedures from three different surgery typesies.

Terminology

Matter:

- Anything that has mass, takes up space and can be clearly identified.
- Examples: tissue, surgical tools, blood
- Counterexamples: reflections, digital overlays, movement artifacts, smoke

Medical instrument to be detected and segmented:

- Elongated rigid object put into the patient and manipulated directly from outside the patient
  - Examples: grasper, scalpel, (transparent) trocar, clip applicator, hooks, stapling device, suction
  - Counterexamples: non-rigid tubes, bandage, compress, needle (not directly manipulated from outside but manipulated with an instrument), coagulation sponges, metal clips

Tasks

Participating teams may enter competitions related to the following tasks:

<u>Binary segmentation:</u>

- Input: 250 consecutive frames (10sec) of a laparoscopic video with the last frame containing at least one medical instrument
- Output: a binary image, in which "0" indicates the absence of a medical instrument and a number ">0" represents the presence of a medical instrument.

Multiple instance detection and segmentation:

- 250 consecutive frames (10sec) of a laparoscopic video;
- Output: a binary image, in which “0” indicates the absence of a medical instrument and numbers “1”, “2“, ... represent different instances of medical instruments.

For both tasks, the entire corresponding video of the surgery is provided along with the training data as context information. In the test phase, only the test image along with the preceding 250 frames is provided.

Labeling instructions

Annotators have access to the video frame to be annotated as well as to the video sequence (for both the training and the test phase!). Before making an annotation, they can scroll through the video sequence.

The results of the manual annotation are one or multiple closed contours as illustrated in the examples below. The interior of these contours represent a medical instrument. Everything outside the provided contours is regarded as other matter (not medical instrument) or image overlay. Contours of the same colour in one image represent all visible parts of one instance of an instrument.

The following decisions were made to ensure consistent labels:

1. Algorithm target: Only medical instruments as defined above should be segmented (see examples 4, 6, 10, 13).
2. Occlusion: Each pixel may correspond to exactly one structure. Specifically, the solid/liquid matter that occurs first along the line of sight of the endoscope determines the label. This may result in multiple contours for a single instrument that is occluded by another instrument, blood or tissue, for example. See examples 2 (instrument) and 9 (tissue) below.
3. Transparency: Medical instruments may be transparent. The occlusion rule holds in this case as well. See examples 8 and 14 below.
4. Holes in instruments: Several medical instruments feature holes (see example 3 below). A hole is made up of pixels that do not show parts of the instrument but are either a) completely surrounded by pixels of the same instrument or (b) are completely surrounded by pixels of one instrument and the margin of the image where it is known, from video context, that the instrument would close the hole outside the image. Following recommendations of previous challenges and given the difficulties of localizing these holes, they are regarded as part of the instrument (i.e. “inpainted”), as illustrated in example 3. The sole exception are trocars when the camera is placed inside of them (see example 14).
5. Text overlay: Text overlay (see example 17) shall be ignored.
6. Image overlay: Image overlays (see example 11 and 16) are treated like “other matter” (not part of instrument), i.e., they are regarded as the first object in the line of light. This also applies for the censor boxes in examples 2 and 5.

Tricky examples

#	Image	How to
1	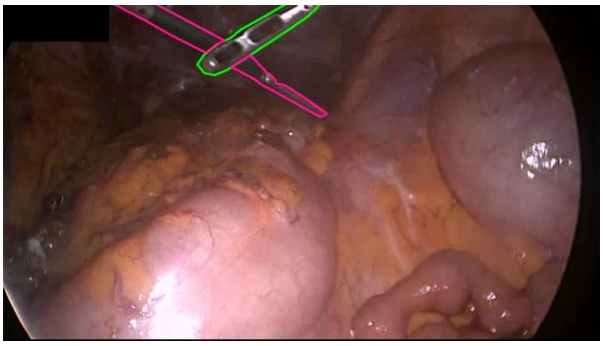	Each pixel may correspond to exactly one structure; the solid/liquid matter that occurs first along the line of sight of the endoscope determines the label (rule 2). In this case, this results in two contours for the instrument with the pink contour.<br>Even if instruments (pink) are visible through holes of another instrument (green), they are not annotated as holes but are regarded as part of the (other) instrument (rule 4).
2	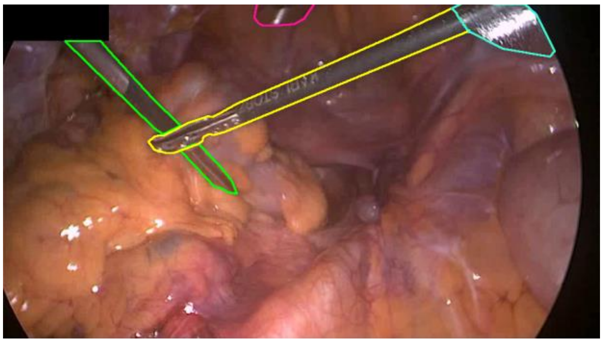	This image contains four instances of medical instruments. The instrument with the green contour is partially occluded by an image overlay (black box - rule 6) and another instrument (yellow). The yellow one is partially occluded by a trocar (rule 2).
3	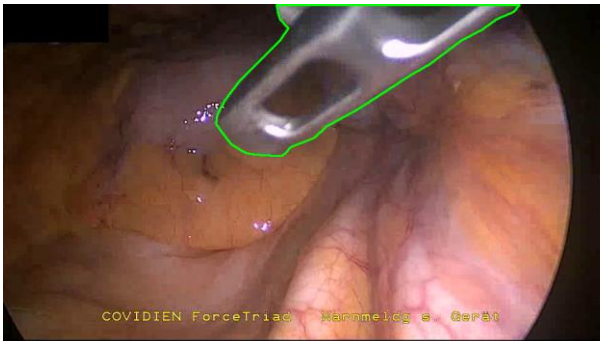	The two holes (one barely visible in the corner) of the medical instrument are regarded as part of the instrument (rule 4).
4	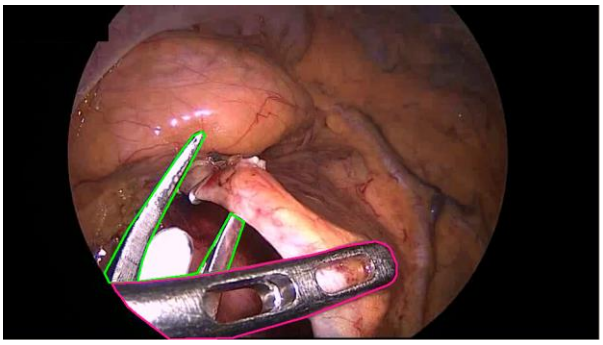	This example does not only show another application of rule 1 and 4 but also emphasizes the distinction of medical instruments and other objects in rule 1 (the white plastic object is not part of an instrument).

5	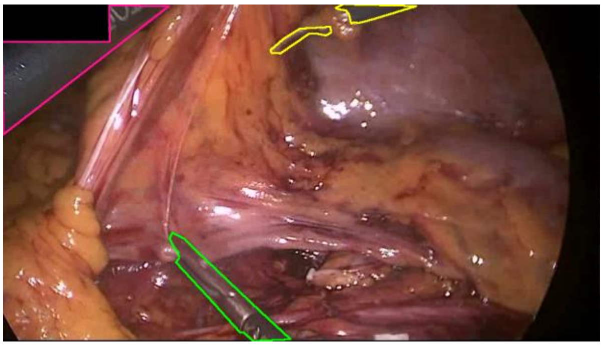	Three instances of medical instruments, two of which are partially occluded (rules 2 and 6).
6	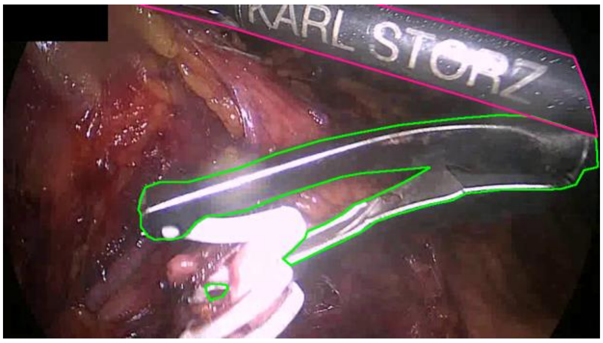	Plastic clamps are not regarded as medical instruments and therefore not annotated (rule 1).
7	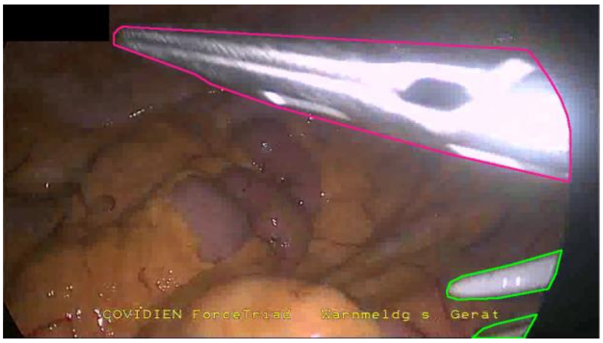	Meta knowledge/video context information was used to conclude that the two object parts in the lower right corner correspond to one instrument.
8	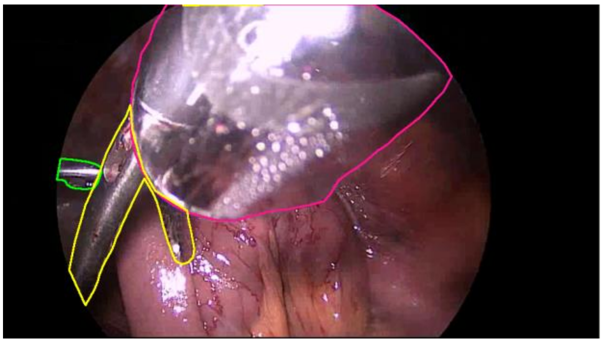	Very challenging image. The transparent trocar occludes part of the instrument represented by the yellow contour (rule 3).
9	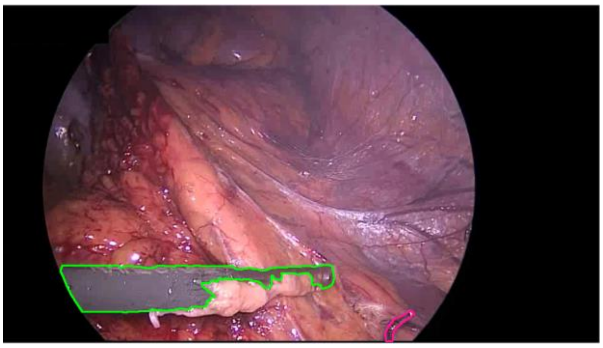	Only visible parts of the instruments are annotated. The instrument corresponding to the green contour is occluded by tissue (rule 2). The one represented by pink is barely visible.

10

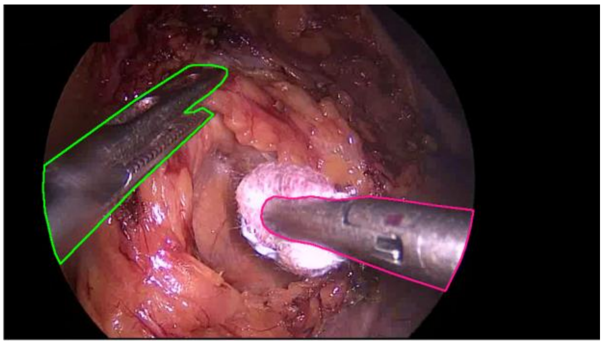

Additional materials that are not regarded as medical instruments (here: bandage) are not segmented (rule 1).

11

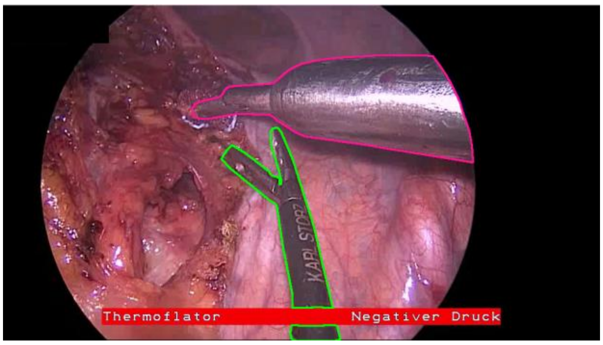

The instrument represented by the green contour comprises two parts due to image overlay (rule 6).

12

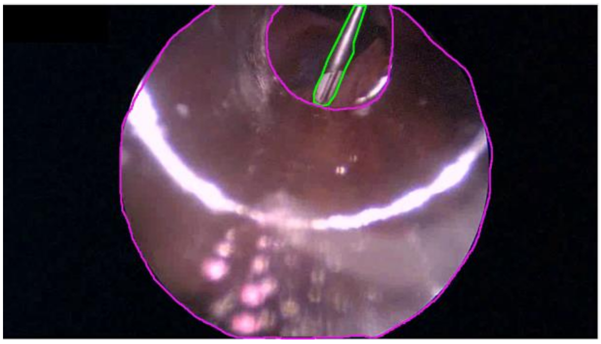

Tricky example. A medical instrument is visible at the end (opening) of the trocar.

13

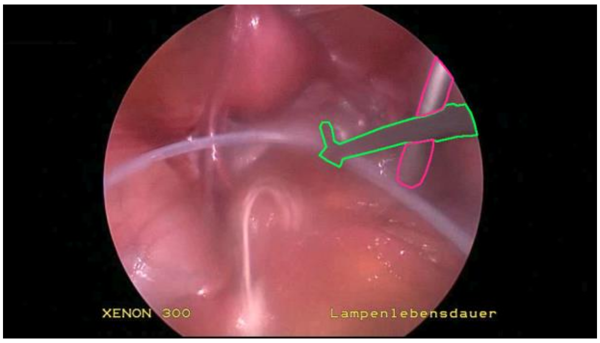

Additional materials that are not regarded as medical instruments (here: drain - plastic) are not segmented (rule 1).

14

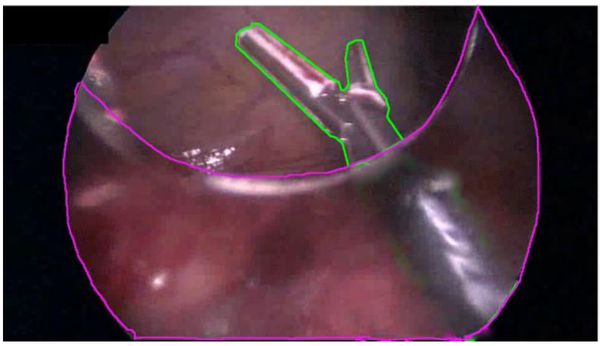

Tricky example. A medical instrument is visible at the end (opening) of the trocar. The part covered by the transparent trocar is not regarded as a visible part of the instrument (rule 2 an 3).

15	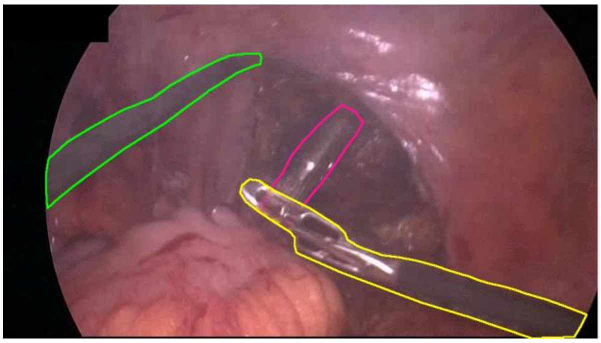	Even if instruments (pink) are visible through holes of another instrument (yellow), they are not annotated because the holes are regarded as part of the (other) instrument (rule 4).
16	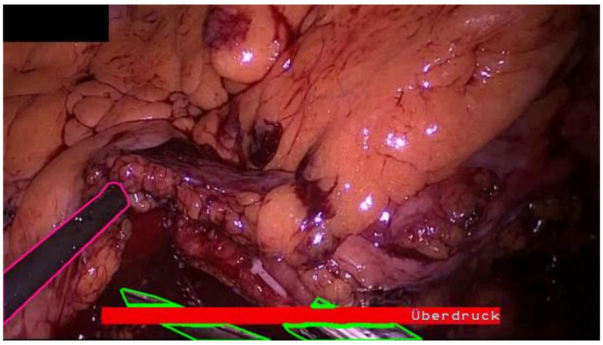	The instrument represented by the green contour comprises multiple parts due to image overlay (rule 6).
17	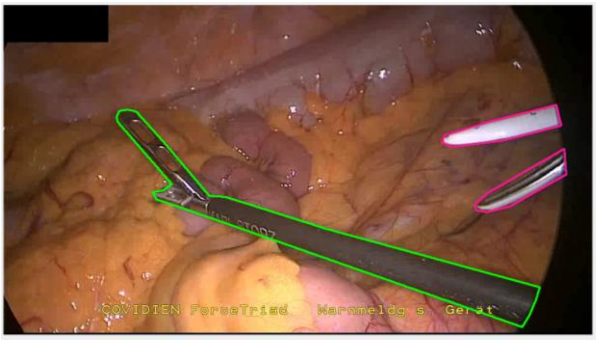	As text overlays only block small areas of sight and form no matter in line of sight, they are ignored for the segmentation (rule 5).
18	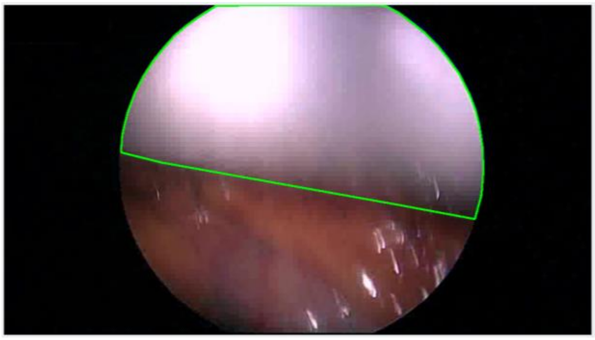	Instruments are visible from a variety of perspectives. The video sequence helps identify blurred close-ups.
19	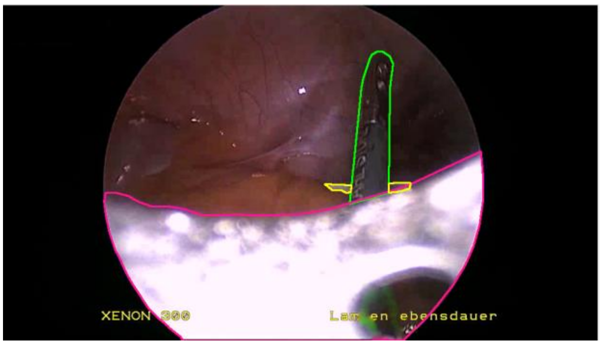	Even if instruments (green) are visible through holes of another instrument (pink), they are not annotated because holes are regarded as part of the (other) instrument (rule 4).

20	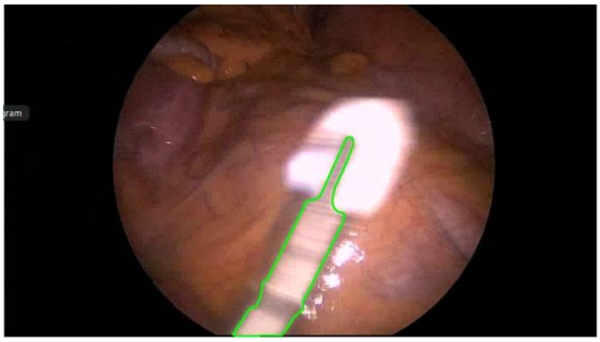	Motion artefacts pose a challenge to accurate and precise annotation. In this example, the inner part of the blurred area has been identified as instrument.

Supplementary Tables

Supplementary Table 1: Example of Device Data

Endoscope	*Thermo-flator*							*OR lights*			*Endoscopic light source*	*Endoscope*		
**Frame #**	**Current gas flowrate**	**Target gas flow rate**	**Current gas pressure**	**Target gas pressure**	**Used gas volume**	**Gas supply pressure**	**Device on?**	**All lights off?**	**Intensity light 1**	**Intensity light 2**	**Intensity**	**White balance**	**Gains**	**Exposure index**
0	115	160	9	15	42	670	0	0	100	100	5	0	-1	-1
1	115	160	9	15	42	670	0	0	100	100	5	0	-1	-1
2	115	160	9	15	42	670	0	0	100	100	5	0	-1	-1
3	115	160	9	15	42	670	0	0	100	100	5	0	-1	-1
4	115	160	9	15	42	660	0	0	100	100	5	0	-1	-1
5	115	160	9	15	43	660	0	0	100	100	5	0	-1	-1
6	115	160	9	15	43	660	0	0	100	100	5	0	-1	-1
7	115	160	9	15	43	660	0	0	100	100	5	0	-1	-1
8	115	160	9	15	43	650	0	0	100	100	5	0	-1	-1
9	115	160	9	15	43	650	0	0	100	100	5	0	-1	-1
10	115	160	9	15	43	650	0	0	100	100	5	0	-1	-1
11	115	160	9	15	43	650	0	0	100	100	5	0	-1	-1
12	115	160	9	15	43	650	0	0	100	100	5	0	-1	-1
13	115	160	9	15	43	650	0	0	100	100	5	0	-1	-1
14	115	160	9	15	43	650	0	0	100	100	5	0	-1	-1
15	115	160	9	15	44	650	0	0	100	100	5	0	-1	-1
16	115	160	9	15	44	650	0	0	100	100	5	0	-1	-1
17	115	160	9	15	44	650	0	0	100	100	5	0	-1	-1
18	115	160	9	15	44	650	0	0	100	100	5	0	-1	-1
19	115	160	9	15	44	650	0	0	100	100	5	0	-1	-1
20	115	160	9	15	44	650	0	0	100	100	5	0	-1	-1
21	115	160	9	15	44	650	0	0	100	100	5	0	-1	-1
…	…	…	…	…	…	…	…	…	…	…	…	…	…	…
371583	30	190	13	19	137	650	0	0	100	100	70	0	-1	-1
371584	30	190	13	19	137	650	0	0	100	100	70	0	-1	-1
371585	30	190	13	19	137	650	0	0	100	100	70	0	-1	-1
371586	30	190	13	19	137	650	0	0	100	100	70	0	-1	-1
371587	30	190	13	19	137	650	0	0	100	100	70	0	-1	-1
371588	30	190	13	19	138	650	0	0	100	100	70	0	-1	-1
371589	30	190	13	19	138	660	0	0	100	100	70	0	-1	-1
371590	30	190	13	19	138	660	0	0	100	100	70	0	-1	-1
371591	30	190	13	19	138	660	0	0	100	100	70	0	-1	-1
371592	30	190	13	19	138	660	0	0	100	100	70	0	-1	-1
371593	30	190	13	19	138	660	0	0	100	100	70	0	-1	-1
371594	30	190	13	19	138	660	0	0	100	100	70	0	-1	-1
371595	30	190	13	19	138	660	0	0	100	100	70	0	-1	-1
371596	70	190	15	19	138	670	0	0	100	100	70	0	-1	-1
371597	70	190	15	19	138	670	0	0	100	100	70	0	-1	-1
371598	70	190	15	19	138	670	0	0	100	100	70	0	-1	-1
371599	70	190	15	19	138	670	0	0	100	100	70	0	-1	-1
371600	70	190	15	19	138	650	0	0	100	100	70	0	-1	-1
371601	70	190	15	19	138	650	0	0	100	100	70	0	-1	-1
371602	70	190	15	19	138	650	0	0	100	100	70	0	-1	-1

Supplementary Table 2: Example of Phase Annotations

Frame #	Phase ID
0	0
1	0
2	0
…	…
11838	7
…	…
17141	3
…	…
37025	7
…	…
48726	6
…	…
73043	4
…	…
82901	5
…	…
85844	6
…	…
102425	4
…	…
118159	8
…	…
124054	1
…	…
133084	8
…	…
147349	4
…	…
152145	1
…	…
156043	4
…	…
159385	8
…	…
169154	0
…	…
172143	8
…	…
232589	9
…	…
310724	10
…	…
340328	11
…	…
369387	12
…	…
371602	12
